# Lecture on the Clinical Examination of Children

**Published:** 1873-05

**Authors:** D. N. Kinsman

**Affiliations:** Professor of Diseases of Women and Children, Starling Medical College, Columbus, O.


*Lecture on the Clinical Examination of Children.* By D. N. KINSMAN, A.M., M.D., Professor of Diseases of Women and Children, Starling Medical College, Columbus, O.

Patience and care are required in the clinical examination of sick children. They cannot talk in most instances, and you must learn to read the *sign language* of disease.

This language is at once clear and explicit, as well as truthful, a statement which we cannot make, without very decided modifications, concerning the oral description of symptoms by adults.

When once you have learned to appreciate the objective signs or symptoms of disease, among children, you will delight more in the investigation of their diseases than those of adults. They are easily frightened, and this disorders circulation and respiration, hence you cannot commence the examination of a sick child abruptly upon your entrance into its room. There are, however, many things which you can study without contact with the child while it is becoming accustomed to your presence. You can observe the color of the skin. This is waxy in atrophy, tuberculosis and wasting diseases; yellow in icterus and post-natal discoloration. There are irregular patches of purplish hue in meningitis, dependent upon diminished power of the vaso-motor nerves; these are produced on the cheek, forehead and neck by pressure of the pillow or the nurse's arm. There is a general congestion of the face in some cases of typhoid fever in its early stages. A circumscribed patch is seen on the cheek in pneumonia and in hectic fever dependent upon tuberculosis or collections of pus. In pneumonia the patch is livid, in hectic, pink. The skin is leaden in color or blue in chills, livid in croup, capillary bronchitis, œdema of the lungs, and all diseases of imperfect aeration of the blood. A similar color is seen in cyanosis from whatever cause. There is paleness in nausea and shock. The "tache cerebrale," which is deemed pathognomonic of meningitis by Trousseau, may be brought out by a simple scratching of the skin, by the finger nail or a pencil. This is dependent upon the same cause as the irregular mottling of the cheek above described. Vogel attaches no value whatever to this sign, but I have seen it brought out in all its characteristics on numerous patients. The redness to which this name is applied persists for a considerable time after the application of the irritation, and I have never been able to produce it except in meningeal inflammation. There is also the white stripe, which may be produced upon the skin by similar means in scarlatina. There are also peculiar eruptions, which you will learn to recognize, in scarlatina, measles, erysipelas and variola.

The rose-colored spots of typhoid fever, the petechiæ of typhus, scorbutus, and epidemic cerebro-spinal meningitis, are often of great value in directing to a correct diagnosis.

In chronic diarrhœa the skin becomes of an earthy hue.

In cases of sudden fever from various causes the skin in children, after the lapse of hours, becomes covered with sudamina. These are caused by a restoration of cutaneous exhalation; the fluid cannot escape through the scarf skin and it accumulates beneath, raising it in the form of minute vesicles. This symptom is of no importance, except that it shows a relaxation of the skin, and an attempt on the part of nature to resume a normal function, which has for a time been arrested.

The eyes of a child when asleep, in health, are directed upward beneath the upper lid, and the pupils are evenly contracted. The pupils may be dilated, irregular or sluggish in their action from cerebral disease, or from disease located in the structure of the eye itself. They are often dilated to a great extent in the early stage of typhoid fever, and when this occurs it shows that the nervous system is profoundly implicated. Dilation occurs also in the later stages of diarrhœas, when there is great exhaustion. The eyelids are also partially open during sleep, in the later stages of exhausting diseases, as the result of loss of muscular tonicity in the orbicularis muscles. In the same cases there is an accumulation of sebaceous matter over the cornea, and a great loss of sensibility, for flies may crawl over the eye without any apparent inconvenience. These symptoms are indicative of great danger.

There is photophobia in meningeal or cerebral disease, also in phlyctenular conjunctivitis. Tears make their appearance about the fourth month, they disappear during severe disease, and their reappearance is an indication of improvement.

Respiration in diseases of the lungs becomes more frequent. The normal rate of respiration in children, when asleep and at rest, is about thirty per minute; when awake it sometimes rises as high as eighty per minute. The mere rate of respiration, since it is subject to so great variation, is not of any special symptomatic value. Respiration, however, is interrupted in cerebral disease, and is a symptom of great value. In croup, inspiration is noisy; in asthma and emphysema, expiration is noisy. Respiration is sighing and slow in nausea.

Cough is another symptom to be referred to the respiratory organs. It is hoarse and ringing in the commencement of croup, becoming extinguished as the disease advances; spasmodic and subintrant in pertussis; constant and synchronous with each expiration in some cases of irritation of the laryngeal nerves.

Cough sometimes exists as a symptom of worms in the intestines, and of jaundice; in these cases it is of reflex origin.

The cry of children has been minutely studied by Billard, and, while I cannot subscribe to all he has written upon the subject, there are two cases in which the cry has diagnostic value. Skoda, I think, first clearly pointed out the difference of the cries in the delirium of typhoid fever and cerebro-spinal meningitis. In typhoid fever the cries are those of constantly changing fancies, and may be changed by external impressions, while in meningitis the cry is a constant repetition of the same word, at intervals more or less regular, with an unvarying cadence.

There is also a class of symptoms belonging to the motor system. The movements of the hand to the head, the ear, the mouth, the throat, are indications of disturbance in the several localities mentioned. In some cases of cerebral irritation and typhoid fever I have observed that the hands are kept constantly in contact with the genitals, and I have learned to regard it as a grave symptom, and that to a great extent it is involuntary.

Jactitation occurs sometimes towards the close of exhausting diseases.

The persistent flexion of one extremity points to lesion in the brain. Flexion of the thumbs or toes, contractions of the eyebrows, grinding of the teeth, and startings, are often the prodromes of general convulsion. Contraction of the lower extremities, with crying, writhing and twisting of the body, are symptoms of the colic, vesical irritation, rectal tenesmus, pricking of pins, etc., and a constant pulling at the penis in young boys, sometimes is seen in calculous disorders, and in congenital phymosis.

There is retraction of the head in meningeal disease, irregular muscular contraction without loss of consciousness in chorea, boring of the head into the pillow in cerebral irritation and rachitis.

Apathy and quietude in a child are suggestive of rachitis when there are no other indications of disease, and when this is joined to sweating about the head and general soreness, the diagnosis is positive.

Nearly all the above symptoms may be made out without touching the child, and many of them can be ascertained without waking it, if it is asleep, and you should never wake a child till you have noted the pulse, respiration and temperature, if you find it asleep.

The frequency and force of the pulse in young children has not the value for us that it has in the case of adults. It is very difficult for us to ascertain the average. An intermittent pulse points with great certainty to disease of the brain, and an extremely frequent and feeble pulse is the forerunner of dissolution.

With the digestive organs many symptoms of value are connected. Vomiting may be incidental to the conformation of the stomach, or a symptom of disease. It is one of the first symptoms of scarlatina, variola or intussusception; it accompanies abdominal inflammations, whooping cough, and sometimes pneumonia. It is one of the most rebellious symptoms of meningeal inflammation; in this disease it is forcible, and has been compared to the action of a force pump. The abdomen is tumid and distended in diarrhœa, but retracted and boat-shaped in meningitis. It fluctuates in dropsy and purulent collections in the peritoneal cavity, is nodular from enlargement of mesenteric glands. In cases of intussusception the coils of the intestines roll beneath the surface like a mass of writhing snakes.

The stools should be carefully examined, for from them you can often derive indications for treatment.

The presence of undigested masses of casein or other albuminous matter tells you the disorder is in the stomach digestion. Excessive watery discharges in summer point to sympathetic paralysis. Worms and their ova will also indicate to you the necessity for their expulsion.

Examine the mouth and fauces, for evidence of disease, for it is in this locality where we commonly first recognize the presence of pseudo-membranous and diphtheritic deposits. The urine should be examined for sugar, albumen and urinary deposits; also for casts of tubes, and renal and vesical epithelium.

There are many things to be learned by inspection, and in obscure troubles it should never be neglected. Needles have been found driven into the brain through the fontanelles, perforating the chest and the abdomen, and plunged into the liver.

One of the earliest evidences of diseased action is found in variations of temperature. The mean temperature of newly born children is a little less, say one-half a degree, than in adults. In scleremia there is a reduced temperature from the beginning.

The production of heat in excess of the natural standard is the result of several factors. There may be increased metamorphosis of tissues; impressions upon the vaso-motor nerves, and the action of poisons upon the blood, as in zymotic diseases, where we infer an action similar to a ferment—all these may be capable of modifying the heat-producing processes; but the subject as yet is to be more fully investigated before we can be fully enlightened. This much we know, there seems to be a fully established law that according to the height of the temperature above 98.40° (Fah.), the gravity of the case and its danger is increased. Adults may have a range of twelve degrees. In children, Roger has recorded oscillations amounting to over eighteen degrees. In intermittent fever there is a great rise of temperature during the febrile paroxysm, often to 104° or 106°, but it speedily begins to decline. In typhoid fever the temperature rises to 102° early in its course, and then by about half a degree or a degree to 104°, which point it does not often pass in children, unless there are complications in the lungs or peritoneal cavity. In diseases of the respiratory organs, when the parenchyma of the lungs is affected, the temperature is notably higher than when the mucous membrane alone is affected. In tubercular meningitis there are great ranges, as well as irregularities in the course of the temperature; the maximum recorded is 108.5°, the minimum 95°. When the substance of the brain is affected, the rise scarcely ever exceeds 101°. A pulse rate increased to 130 or more per minute and a temperature of 102° is prognostic of meningitis, while a pulse rate of 110 to 120, with a persistent temperature of 104°, points to typhoid fever as the disease.

These general facts will enable you to perceive something of the importance of a study of the temperature. For its full discussion I must refer you to the work of Seguin, and to the works on practice of medicine.

Auscultation and percussion should be practiced in cases of suspected disease of the lung. Auscultation first, and usually you can learn all you wish to know by placing the ear to the back of the chest. The general signs are the same as in adults, except that the vesicular murmur is more distinct or "puerile." By crying and expulsive movements the child can extend the range of dullness fully three inches, by the raising of the lower and abdominal organs against the diaphragm. Remember this, or you will diagnose a pneumonia in a crying child when none exists.

Percussion has not the value in children which it has in adults, because they are more restless, and by their crying obscure all true perception of percussion sounds.—*Kansas City Med. Journal.*

